# Mixed-Phenotype Acute Leukemia Transforming Into Acute Myelomonocytic Leukemia (AML M4): A Case Report and Therapeutic Challenges

**DOI:** 10.7759/cureus.96576

**Published:** 2025-11-11

**Authors:** Bakr Alhayek, Xiaowei Malone, Tamim Rabie, Ryan Brink, Raja Gummalla

**Affiliations:** 1 Internal Medicine, AdventHealth Tampa, Tampa, USA; 2 General Surgery, Maria Hilf Hospital, Krefeld, DEU

**Keywords:** clonal evolution, lineage switch relapse, minimal residual disease, mixed-phenotype acute leukemia, myeloid transformation, precision oncology

## Abstract

Mixed-phenotype acute leukemia (MPAL) accounts for a fraction of de novo acute leukemias and carries a dismal prognosis, especially when adverse lesions such as monosomy 7 are present. Consensus on optimal frontline therapy remains lacking. Herein, we present a 69-year-old woman who presented with pancytopenia and 79% circulating blasts. Immunophenotyping and cytogenetics established T/myeloid MPAL with a hypodiploid clone 45, XX, dic(7;12)(p11.2;p13)(19)/46,XX and an *IDH2* R140Q mutation. Hyperfractionated cyclophosphamide, vincristine, doxorubicin, and dexamethasone (Hyper-CVAD) induction achieved morphological complete remission, but measurable residual disease (MRD) persisted. Consolidation with mini-cyclophosphamide, vincristine, and dexamethasone (mini-CVD) and prednisone, vincristine, methotrexate, and 6-mercaptopurine (POMP) maintenance failed to eradicate MRD, and overt relapse occurred at month 7. Nelarabine salvage was initiated. After two nelarabine cycles (month 8.5), bone marrow contained 85% myelomonocytic blasts. Cytogenetic evolution to 45,XX,psu dic(7;12)(p11.2;p11.2)/45,idem,del(16)(q12) marked transformation to acute myelomonocytic leukemia (AML-M4). Profound pancytopenia led to invasive pulmonary aspergillosis and symptomatic severe acute respiratory syndrome coronavirus 2 (SARS-CoV-2) infection. With refractory disease and escalating infectious morbidity, active therapy was discontinued; the patient died 11 months after diagnosis. This case illustrates rapid clonal evolution of T/myeloid MPAL into chemoresistant AML-M4 driven by persistent chromosome-7 loss and acquisition of del(16q). Early molecular risk stratification and deployment of targeted agents, venetoclax-based combinations, or timely allogeneic transplantation should be considered before irreversible genomic complexity emerges. Prospective studies tailored to high-risk cytogenetic subsets of MPAL are urgently needed.

## Introduction

Mixed-phenotype acute leukemia (MPAL) is a biologically heterogeneous, yet clinically aggressive form of acute leukemia characterized by either a single leukemic clone expressing lineage-defining markers of both acute lymphoblastic leukemia (ALL) and acute myeloid leukemia (AML), or by two immunophenotypically distinct blast populations each meeting the diagnostic criteria for a different lineage [1]. Since its initial recognition as a distinct entity in the 2008 World Health Organization (WHO) classification, successive revisions - most recently the fifth edition published in 2022 - have refined the immunophenotypic criteria and introduced genotype-defined subgroups such as BCR-ABL1-positive and KMT2A-rearranged MPAL [2,3]. Contemporary registry data indicate that MPAL accounts for roughly 2-3% of adult and up to 5% of pediatric de novo acute leukemia cases [3].

The diagnosis of MPAL requires identification of either a single blast population with lineage-defining markers of multiple lineages (biphenotypic) or two distinct blast populations, each satisfying the diagnostic criteria for a different lineage, for example, one meeting AML criteria and another meeting B-lymphoblastic leukemia (B-ALL) or T-lymphoblastic leukemia (T-ALL) criteria, representing a bilineage presentation [1,2]. Recent genomic studies have revealed distinct mutational and methylation signatures in MPAL that are associated with differences in the expression of lineage commitment genes [4]. Common genetic abnormalities in MPAL include complex karyotypes (CKs), BCR-ABL1 translocations, and mutations in genes such as RUNX1 and FLT3-ITD [1,3].

Because MPAL is rare, no standardized treatment regimen has been established, and management remains empirical. Retrospective series support an acute lymphoblastic leukemia (ALL)-type induction therapy - typically hyperfractionated cyclophosphamide, vincristine, doxorubicin, and dexamethasone (Hyper-CVAD) or a pediatric-inspired regimen - followed by allogeneic hematopoietic cell transplantation in first complete remission (CR), when feasible [5,6]. The American Society of Clinical Oncology (ASCO) and the College of American Pathologists (CAP) jointly recommend comprehensive genomic testing at diagnosis to identify actionable lesions (e.g., FLT3-ITD, IDH1/2 mutations, BCR-ABL1 rearrangements) and to inform the timing of transplantation [4].

Here, we describe an adult case of T/myeloid MPAL harboring dic(7;12) and partial monosomy 7 that rapidly evolved into acute myelomonocytic leukemia (AML-M4) with the acquisition of del(16q).

## Case presentation

A 69-year-old woman with a history of hypertension and a remote, conservatively managed subdural hematoma presented with a three-week history of cervical‐node enlargement, exertional fatigue, and easy bruising. Initial blood work (Table 1) revealed pancytopenia-hemoglobin 7.4 g/dL, absolute neutrophil count (ANC) 0.6 × 10^9^/L, platelets 18 × 10^9^/L. Peripheral blood flow cytometry (images could not be obtained from the originating facility) showed 80% blasts that were cCD3⁺/CD7⁺/CD34⁺/CD117⁺/HLA-DR⁺ but myeloperoxidase-negative, consistent with acute leukemia of ambiguous lineage. Bone-marrow aspirate obtained the same week contained 80% blasts and an 80% cellular marrow.

**Table 1:** Baseline laboratory evaluation WBC: white blood cell count; ANC: absolute neutrophil count; LDH: lactate dehydrogenase; Tbili: total bilirubin; PT/INR: prothrombin time/international normalized ratio; aPTT: activated partial thromboplastin time; TLS: tumor lysis syndrome

Test	Result (presentation)	Units	Reference interval	Interpretation
Hemoglobin	7.4	g/dL	12.0-16.0	Severe anemia
WBC	15.97	×10^9^/L	4.0-10.0	-
Blasts (peripheral blood)	80	% of WBC	0	Markedly increased
ANC	0.6	×10^9^/L	1.5-7.0	Severe neutropenia
Platelets	18	×10^9^/L	150-400	Severe thrombocytopenia
LDH	297	U/L	135-225	Tumor lysis/turnover marker
Uric acid	4.6	mg/dL	2.6-6.0	TLS risk assessment
Creatinine	0.7	mg/dL	0.6-1.1	Renal function
AST/ALT	12/12	U/L	AST 10-40/ALT 7-56	Hepatic function
Tbili	0.9	mg/dL	0.2-1.2	-
PT/INR	15.7/1.15	s/-	11-13.5/0.8-1.2	Coagulation
aPTT	30.4	s	25-35	Coagulation
Ferritin	9986	ng/mL	13-150	Inflammation/iron
HBsAg/anti-HBc/HCV Ab/HIVAg/Ab	Negative	-	Negative	Pre-treatment screening

Conventional karyotyping disclosed a hypodiploid clone, 45,XX,dic(7;12)(p11.2;p13)(19)/46,XX(1); targeted next-generation sequencing identified an IDH2 R140Q mutation, whereas FLT3-ITD/TKD and IDH1 were negative. Transthoracic echocardiography documented a left-ventricular ejection fraction of 70% with moderate tricuspid regurgitation. These findings satisfied the WHO 2022 criteria for T/myeloid MPAL.

Hyper-CVAD cycle 1A was initiated immediately and achieved count recovery at week 6. Bone marrow showed morphologic CR but measurable residual disease (MRD). Mini-cyclophosphamide, vincristine, and dexamethasone (mini-CVD) consolidation began in week 7, but two admissions for extended-spectrum β-lactamase (ESBL) - *Klebsiella *bacteremia and prolonged grade-4 cytopenias - followed. Persistent MRD at week 18 prompted conversion to outpatient POMP (prednisone, vincristine, 6-mercaptopurine, low-dose methotrexate) with antiviral, antibacterial, and antifungal prophylaxis and twice-weekly laboratory monitoring. Key hematologic parameters across these milestones are summarized in Table 2.

**Table 2:** Clinical, hematologic, and genomic milestones Hb: hemoglobin; ANC: absolute neutrophil count; MRD: measurable residual disease; CR: complete remission; CNV: copy-number variation; neg: negative; MPAL: mixed-phenotype acute leukemia; AML-M4: acute myelomonocytic leukemia; Hyper-CVAD: hyperfractionated cyclophosphamide, vincristine, doxorubicin, and dexamethasone; FISH: fluorescence in situ hybridization

Milestone/Time-point	Hb (g/dL)	ANC (×10^9^/L)	Platelets (×10^9^/L)	Marrow status	Karyotype (metaphases)	FISH/CNV	Key molecular findings	Interpretation
Presentation (Month 0)	7.4	0.6	18	80% blasts	45,XX,dic(7;12)(p11.2;p13)(19)/46,XX(1)	-	IDH2 R140Q; FLT3 ITD/TKD neg; IDH1 neg	Pancytopenia with high blast burden; hypodiploid clone with dic(7;12) and IDH2 mutation establishes high-risk T/myeloid MPAL.
Post-hyper-CVAD (Week 6)	10.3	1.8	162	MRD-positive CR	-	-	-	Hematologic recovery and morphologic CR, but persistent MRD indicates incomplete clearance and high relapse risk.
Post-mini-CVD (Week 18)	9.8	1.2	96	MRD-positive	-	-	-	Maintained remission with partial count recovery, but continued MRD suggests a refractory clone despite consolidation.
Relapse (Month 7)	6.9	0.2	9	74% blasts	-	-	-	Clinical and morphologic relapse with severe pancytopenia, confirming treatment resistance.
Post-nelarabine ×2/AML-M4 (Month 8.5)	7.1	0.1	11	85% AML-M4	45,XX,psu dic(7;12)(p11.2;p11.2)(6)/45,idem,del(16)(q12)	CBFB/16q loss in 73%	FLT3 ITD/TKD neg; NPM1 neg; IDH2 not re-tested	Lineage switch to AML-M4 with new del(16q) on background of dic(7;12), reflecting clonal evolution and chemoresistance.

Despite this, frank relapse emerged in month 7 (hemoglobin 6.9 g/dL, ANC 0.2 × 10^9^/L, platelets 9 × 10^9^/L, 74% circulating blasts). POMP was discontinued, and nelarabine 1,500 mg/m^2^ intravenously on days 1, 3, 5 of a 21-day cycle was started. Anticoagulation for a recent lower-extremity deep vein thrombosis was withheld whenever platelets fell below 50 × 10^9^/L. Serial cerebrospinal fluid examinations remained negative for blast, and Epstein-Barr virus polymerase chain reaction (PCR) was negative.

**Figure 1:**
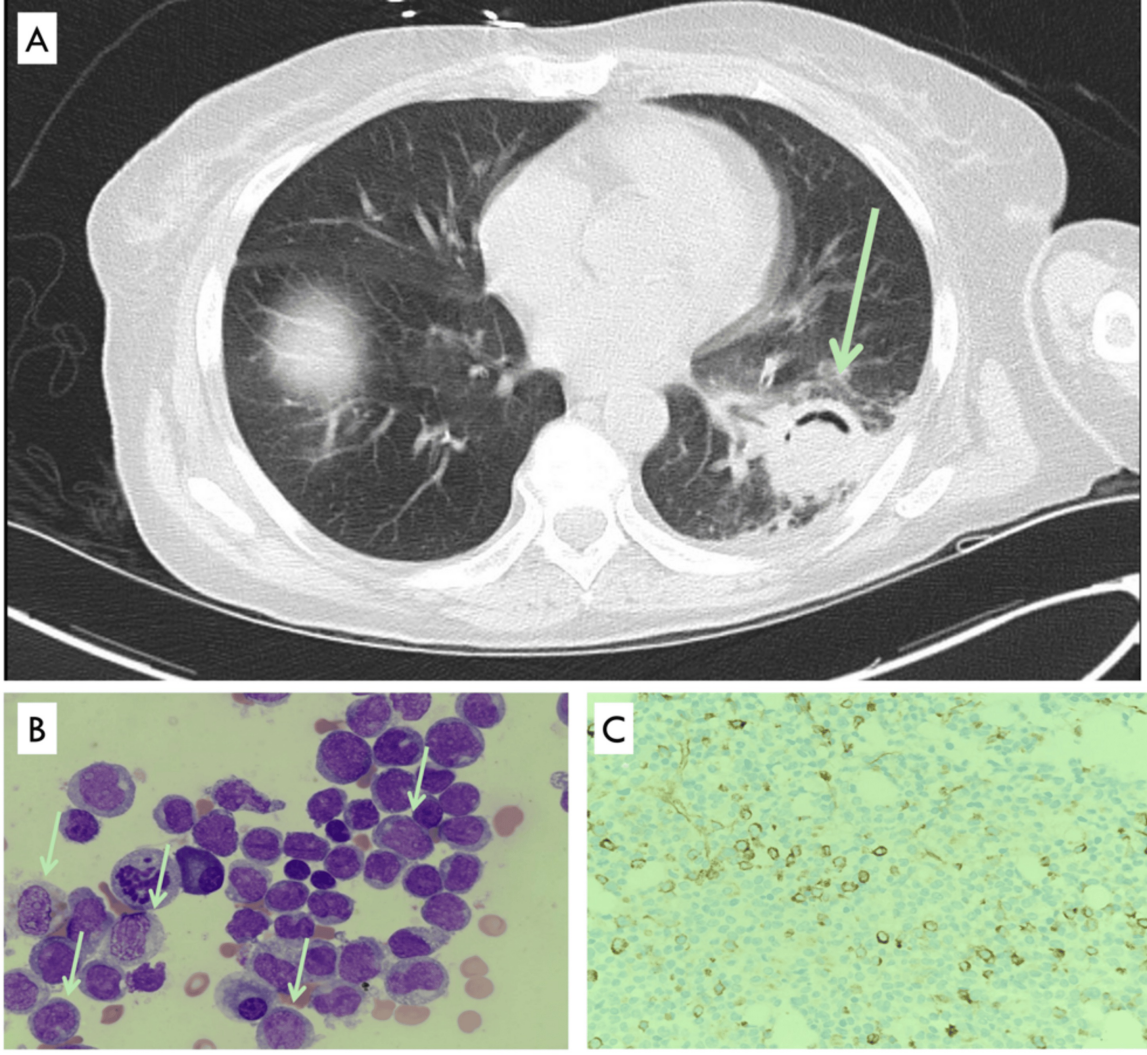
Pulmonary and marrow findings at leukemic transformation (A) Chest CT demonstrates a thick-walled cavitary lesion with an air-crescent sign (green arrow) in the left lower lobe, typical of invasive pulmonary aspergillosis. (B) Bone-marrow aspirate smear (Wright-Giemsa, ×1,000) shows numerous blasts with fine chromatin, prominent nucleoli, and basophilic cytoplasm (arrows). (C) Immunohistochemistry for CD34 on the corresponding trephine biopsy highlights strong membranous staining in the blast population, confirming their immaturity.

After two nelarabine cycles (month 8.5), bone marrow contained 85% myelomonocytic blasts (Figures 1B, 1C). Cytogenetics had evolved to 45,XX,psu dic(7;12)(p11.2;p11.2)(6)/45,idem,del(16)(q12); fluorescence in situ hybridization confirmed CBFB/16q loss in 73% of nuclei. FLT3-ITD/TKD and NPM1 remained negative. The findings established transformation to AML-M4 within the original dic(7;12) clone.

Profound pancytopenia precipitated invasive pulmonary aspergillosis and symptomatic SARS-CoV-2 infection (Figure 1A). Platelet counts <10 × 10^9^/L necessitated permanent discontinuation of anticoagulation, although the chronic subdural hematoma remained radiographically stable. Following a goals-of-care discussion, the patient elected best supportive management. Despite near-daily platelet transfusions, she died of septic shock and respiratory failure 11 months after diagnosis.

## Discussion

MPAL represents a diagnostically and therapeutically challenging entity in which a single neoplastic clone exhibits overlapping lymphoid and myeloid features [7]. Although MPAL accounts for fewer than 5% of adult acute leukemias, it is uniformly regarded as high risk because of its frequent association with adverse cytogenetic lesions, persistence of MRD, and propensity for early, therapy-refractory relapse [8].

The present case, characterized at presentation by a dic(7;12)-driven partial monosomy 7 and progressing to acquire del(16q) accompanied by a lineage switch to AML M4, presents an intriguing opportunity to explore four interconnected aspects: (i) the biological mechanisms underlying lineage switching in MPAL; (ii) the prognostic implications of monosomy 7 and evolving cytogenetic complexity; (iii) emerging therapeutic avenues, such as IDH2 inhibition, venetoclax-based regimens, and cellular immunotherapies; and (iv) how this clinical trajectory aligns with, or diverges from, published reports of MPAL transforming into AML.

Lineage switch in acute leukemia is now thought to arise primarily through clonal selection of a pluripotent (multipotent) leukemic stem-cell compartment. In contrast, direct trans-differentiation of mature blasts is regarded as a less common mechanism [9]. Multi-omic single-cell profiling of newly diagnosed adult MPAL shows that, irrespective of immunophenotype, virtually all blasts share a stem-cell-like transcriptional program enriched for self-renewal and multilineage priming, conferring what the authors term "high differentiation potential" [10]. Under therapy, this plastic pool can reseed disease along whichever lineage affords greatest survival [11]. In our patient, lymphoid-directed hyper-CVAD likely suppressed the T-lineage branch, selecting a myeloid-committed subclone that later surfaced as AML M4.

Genetic instability provides the substrate for that selection. The founding dic(7;12) lesion removes large segments of 7q and 12p, regions harboring regulators of hematopoietic differentiation such as IKZF1 and ETV6; loss of these loci has been linked to impaired lineage fidelity and aggressive behavior in lineage-ambiguous leukemias [12-15]. Monosomy 7 in particular has been associated with documented T-to-myeloid switches during or after chemotherapy, underscoring its role in clonal adaptability [16-18]. Subsequent acquisition of del(16q) in our case further destabilized the genome, coinciding with overt myelomonocytic transformation.

Therapeutic pressure amplifies these evolutionary dynamics. Lineage-specific treatments, such as CD19 CAR-T or ALL-type chemotherapy, can eradicate the dominant phenotype while sparing-indeed, favoring the outgrowth of a minor clone along an alternate differentiation trajectory [19,20]. This "escape via immunophenotypic drift" is now recognized as a mechanism of relapse across acute leukemia subtypes [21]. The convergence of an inherently plastic stem-cell transcriptome, high-risk cytogenetic losses deregulating lineage commitment, and intense lineage-directed selection pressures therefore provides a coherent biological explanation for the rapid T-to-myelomonocytic switch observed in this patient [10].

The same genomic forces that permit lineage flexibility also dictate clinical risk. Monosomy 7 is one of the most powerful adverse-risk markers across acute leukemias. Large AML datasets show that isolated monosomy 7 is associated with low remission rates and inferior long-term survival, which deteriorate further when -7 occurs within a CK [22]. CK occurs in approximately one-fifth to one-third of adult MPAL cases﻿ and portends an outcome as poor as that seen in AML or ALL with the same cytogenetic burden [3,23]. In the Bone‑Marrow Pathology Group study of 2023, MPAL with CK accounted for 19-32% of cases and showed overall survival indistinguishable from AML/ALL with CK, irrespective of the chemotherapy lineage chosen [24]. A separate single-center series of 117 adults reported CK in 24% of karyotyped MPAL patients, confirming its place among the most frequent genomic anomalies in this entity [25].

Because MPAL is rare, neither the NCCN nor recent European LeukemiaNet (ELN) publications offer a dedicated treatment algorithm; both documents merely note that most centres adopt an ALL-style induction (e.g., hyper-CVAD) and proceed to allogeneic transplantation in first remission whenever feasible [5].

Early next-generation sequencing can uncover actionable lesions such as IDH2 [26]. In relapsed AML, the selective inhibitor enasidenib achieved a 40% overall response in the phase I/II AG221-C-001 trial and quadrupled remission rates over conventional care in the randomised IDHENTIFY study, albeit without an overall-survival advantage [27].

When no targetable mutation is present, or after IDH-directed therapy, venetoclax-based combinations provide a second pharmacologic lever [28]. Case reports and the five-patient series of Wang et al. document CRs in both newly diagnosed and relapsed MPAL with venetoclax plus hypomethylating agents (HMAs), occasionally permitting successful allo-transplant [28-30]. A transplant-adapted venetoclax/HMA study (NCT04128501) is actively enrolling and explicitly allows MPAL histology, offering a prospective option for candidates unable to tolerate intensive chemotherapy.

Patients who relapse despite small-molecule therapy, or whose blasts express a clear antigenic target, may benefit from CAR-T approaches. Donor-derived CD19 CAR-T cells have produced MRD-negative remissions in B/myeloid MPAL, though myeloid antigen escape remains a risk [31]. CD7-directed CAR-T platforms, engineered to avoid T-cell fratricide, have recently induced durable CRs in CD7-positive T/myeloid MPAL [32]. Dual-target constructs (e.g., CD19 + CD33; NCT05066165) are entering first-in-human testing to mitigate lineage-switch relapse [33].

We performed a focused review of peer-reviewed case reports published from 2010 through 2025 that document lineage conversion in mixed-phenotype or lineage-ambiguous leukemia. The 14 qualifying patients (Table 3) [34-47] reveal four consistent patterns. First, lineage switch was almost always precipitated by lineage-directed therapy: eight events followed CD19-targeted blinatumomab, one occurred after CD19 CAR-T cells, one arose during hyper-CVAD induction, and the remaining four followed conventional pediatric chemotherapy. Second, adverse cytogenetics were the rule. Seven of 14 cases (50%) harbored KMT2A rearrangements, whereas the remainder displayed complex or chromosome-7-related lesions, closely paralleling the dic(7;12)/-7 → del(16q) evolution observed in our patient. Third, the terminal phenotype was heavily biased toward monocytic or myelomonocytic AML, supporting the concept that myeloid differentiation represents a favored escape route for pluripotent clones. Finally, prognosis was dismal: durable survival (>24 months) was recorded only in three pediatric patients who proceeded rapidly to allogeneic transplantation, and even those remissions remain short in molecular terms. Taken together, these data indicate that lineage switch in MPAL is not a clinical oddity but a foreseeable manifestation of stem-cell plasticity under intense selective pressure - one that warrants early molecular surveillance, expeditious transplant referral, and enrolment in lineage-agnostic or dual-targeted immunotherapy trials.

**Table 3:** Published lineage-switch events in acute leukemia (2010-2025) and the present MPAL case AMP: amplification; Blina: blinatumomab; CR: complete remission; HiDAC: high-dose cytarabine; MPAL: mixed-phenotype acute leukaemia; mut: mutation; NR: not reported; B-ALL: B-lymphoblastic leukemia; AML: acute myeloid leukemia

First author and year	Age/sex	Initial diagnosis and key genetics	Lineage-directed therapy at switch	Phenotype after switch	Clinical outcome (follow-up)
Imataki et al. 2010 [34]	60 F	Pre-B-ALL; normal karyotype	Post-induction CR (conventional ALL chemo)	AML M5	Refractory; died day 120
Park et al. 2011 (4 paediatric cases) [35]	0.3-7 y (2 F/2 M)	3 × B-ALL, 1 pre-B; 2 MLL-r, 1 hyper-diploid	On- or early off-therapy relapse	AML (M0/M1/M4)	3 long-term CR_2_ (17-79 mo); 1 death
Rossi et al. 2012 (Series of 9) [36]	0.4-9 y (5 F/4 M)	7 B-ALL, 2 bilineal AML; 7/9 MLL-r	Median day 15 of induction	7 × AML M4/M5; 2 pro-B-ALL	All died < 1 y
Grammatico et al. 2013 [37]	25 F	Pro-B-ALL, TAF15-ZNF384	During maintenance (HiDAC salvage)	AML M5b	Induction failure; died 9 mo post-switch
Balducci et al. 2017 [38]	15 M	B-ALL, KMT2A-AFF1	Blinatumomab	Monocytic AML	Refractory; died day 180
Haddox et al. 2017 [39]	40 F	B-ALL, t(4;11), MPL mut.	Blinatumomab	Myelomonocytic AML	No CR; hospice day 140
Zoghbi et al. 2017 [40]	8 F	Common-ALL, hyper-diploid	Blinatumomab post-HSCT	CD19⁻ myeloid blasts	Progressive; outcome NR
Ruiz-Delgado et al. 2017 [41]	60 M	Hyper-diploid pre-B-ALL, BCR-ABL1⁻	Day 60 of TOTAL XI induction	AML (CD13 CD33 CD117⁺)	Active AML at 90 d
He et al. 2019 [42]	40 F	B-ALL, KMT2A-AFF1	Blinatumomab salvage	AML + myeloid sarcoma → B/My MPAL	Multiple relapses; alive CR_2_ at 18 mo
Aujla et al. 2019 [43]	32 F	T-ALL/LBL; ALK, MAP3K14 muts.	Hyper-CVAD	AML → later T-ALL re-switch	Alive; bidirectional plasticity
Zhu et al. 2020 [44]	31 M	T-pro-ALL; no fusion detected	26 mo post-CR_1_ chemo	B-ALL (BCR-ABL1 p210)	Alive CR_2_ at 12 mo
Du et al. 2021 [45]	0.5 F	Infant B-ALL, KMT2A-EPS15	Blinatumomab salvage	Monocytic AML	HSCT → early B-ALL relapse; died
Takeda et al. 2022 [46]	65 F	Ph⁻ B-ALL, complex karyotype, TP53, MLL amp.	Refractory multi-chemo	B/T MPAL → monoblastic AML	Died < 8 mo
Jamil et al. 2023 [47]	10 M	Non-APL AML	First relapse	T-ALL	Died during salvage
Present case 2025	69 F	T/myeloid MPAL; hypodiploid 45,XX,dic(7;12)(p11.2;p13); IDH2 R140Q	Nelarabine salvage (after hyper-CVAD → mini-CVD → POMP)	AML-M4 (myelomonocytic); acquired del(16q)	Refractory; died (11 months)

## Conclusions

Our report extends the small but growing literature on lineage conversion in MPAL by documenting, to our knowledge, the first adult T/myeloid MPAL with dic(7;12) that evolved into AML-M4 after acquiring del(16q). Whereas KMT2A rearrangements drive most published switches, our case underscores that alternative genomic routes, here, combined loss of chromosome-7 material and 16q, can likewise endow a pluripotent clone with the capacity to shift lineage. The rapid, overt relapse after hyper-CVAD and nelarabine highlights the narrow therapeutic window in such patients and argues for early allogeneic transplantation or enrolment in trials of dual-targeted or lineage-agnostic immunotherapies. Taken together with the literature review, our findings reinforce that lineage switch in acute leukemia, though uncommon, portends an aggressive course that is seldom mitigated by standard regimens. Continued case reporting will clarify why monocytic or myelomonocytic phenotypes emerge so frequently and will hopefully accelerate the development of strategies that attack the underlying plastic leukemic stem-cell compartment.
